# Central Projections of Gustatory Receptor Neurons in the Medial and the Lateral Sensilla Styloconica of *Helicoverpa armigera* Larvae

**DOI:** 10.1371/journal.pone.0095401

**Published:** 2014-04-16

**Authors:** Qing-Bo Tang, Huan Zhan, Huan Cao, Bente G. Berg, Feng-Ming Yan, Xin-Cheng Zhao

**Affiliations:** 1 Department of Entomology, College of Plant Protection, Henan Agricultural University, Zhengzhou, Henan, China; 2 Department of Psychology, Neuroscience Unit, Norwegian University of Science and Technology, Trondheim, Norway; AgroParisTech, France

## Abstract

Food selection behavior of lepidopteran larvae is predominantly governed by the activation of taste neurons present in two sensilla styloconica located on the galea of the maxilla. In this study, we present the ultrastructure of the sensilla styloconica and the central projection pattern of their associated receptor neurons in larvae of the heliothine moth, *Helicoverpa armigera*. By means of light microscopy and scanning electron microscopy, the previous findings of two morphologically fairly similar sensilla comprising a socketed conic tip inserted into a large peg were confirmed. However, the peg size of the medial sensillum was found to be significantly bigger than that of the lateral sensillum. The sensory neurons derived from each sensillum styloconicum were mapped separately using anterograde staining experiments combined with confocal laser-scanning microscopy. For determining the afferents’ target regions relative to each other, we reconstructed the labeled axons and placed them into a common reference framework. The sensory axons from both sensilla projected via the ipsilateral maxillary nerve to the suboesophageal ganglion and further through the ipsilateral circumoesophageal connective to the brain. In the suboesophageal ganglion, the sensory projections targeted two areas of the ipsilateral maxillary neuropil, one located in the ventrolateral neuromere and the other adjacent to the neuromere midline. In the brain, the axon terminals targeted the dorso-anterior area of the ipsilateral tritocerebrum. As confirmed by the three-dimensional reconstructions, the target regions of the neural projections originating from each of the two sensilla styloconica were identical.

## Introduction

Insects possess gustatory systems that allow them to discriminate between host plants and non-host plants. In lepidopteran larvae, it has been demonstrated, both via behavioral and electrophysiological studies, that the main taste organ consists of two sensilla stylochonica localized on the maxilla [1]–[6]. The ultrastructure of these sensilla is reported to be conserved across lepidopteran species [5], [7]–[16]. Each sensillum is innervated by five bipolar neurons, i.e., four chemosensory and one mechanosensory [5], [7], [10], [14], [17], [18]. Electrophysiological experiments performed on larvae of more than 20 lepidopteran species have shown that each sensillum responds to both phagostimulants and deterrents, including water, sugars, sugar alcohols, amino acids, salt, and bitter substances [1]–[3], [5], [7], [17], [19]–[30].

Like other sensory modalities, gustatory information is transduced to patterns of nerve impulses being conveyed to particular regions of the central nervous system [4], [6], [31]. The central projections of the gustatory neurons housed by the sensilla stylochonica have previously been mapped in larvae of two lepidopteran species, i.e. the tomato hornworm, *Manduca sexta* (Lepidoptera: Sphingidae) [32], and the Japanese oak silkmoth, *Antheraea yamamai* (Lepidoptera: Saturniidae) [33]. In both species, the sensory axons from the two sensilla were reported to target first the ipsilateral suboesophageal ganglion (SOG) via the maxillary nerve (MxN) and then the ipsilateral brain via the circumoesophageal connectives. None of these studies mapped the target regions according to each sensillum, however. The more thoroughly studied larvae of flies possess gustatory organs including the so-called ventral and terminal organ located on the cephalic lobe [34], [35]. In spite of different morphology of the external taste organs of lepidopteran and dipteran fly larvae, the projection pattern of the associated afferents in the two insect groups are partly similar by axons targeting the ipsilateral region of the SOG via the MxN [4], [35]–[39].

The projection pattern of the sensory axons originating from the two sensilla styloconica of larvae in the Noctuidae, the largest family of Lepidptera, has not been described previously, however. The cotton bollworm, *Helicoverpa armigera* (Hübner) (Lepidoptera: Noctuidae), is a typical polyphagous species feeding on at least 160 plant species [40]–[42]. It has been reported that each of the two sensilla styloconica of *H. armigera* larvae responds to various phagostimulants and plant secondary compounds [1], [26], [29], [43]–[46], and that the larval feeding preference to different host leaves displays heritable traits [47]. In the present study, we have investigated the morphological characteristics of the two sensilla styloconica of this species by means of light microscopy and scanning electron microscopy and mapped the target regions of their sensory neurons by means of confocal microscopy. Importantly, we have labeled neurons housed by each of the two sensilla selectively. As compared to the previous data, obtained from cobalt stainings performed on larvae of the moth species mentioned above, the current study includes high resolution confocal microscopy combined with three-dimensional reconstructions and thus offers the opportunity of precisely comparing the regions of the central nervous system being innervated by the assembly of gustatory afferents originating from each sensillum.

## Materials and Methods

### Insects

The 5^th^ instar larvae of *H. armigera* were used for the experiments. The larvae, which were obtained from the established laboratory colony, were reared on an artificial diet [48], and kept at a temperature of 27°C and 75% relative humidity with a photoperiod regime of 16 h light and 8 h darkness.

### Morphology of the Sensilla

The outer morphology of the sensilla styloconica was examined by a scanning electron microscope (S-3400N II, Hitachi Co, Japan). The excised head of the fifth-instar caterpillar was fixed in 2.5% glutaraldehyde in 0.1 M phosphate buffer (pH 7.4) and then dehydrated with ethanol series. Next, the preparation was mounted on a stub, air-dried, and gold-coated with sputter-coating unit before being examined by scanning electron microscopy.

In order to compare the size of the medial and the lateral sensilla styloconica on the maxillary galea, we measured the length and diameter of each organ in 30 *H. armigera* larvae using a Keyence Digital Microscope (VHX-600, Japan). Since neither the conic tip nor the peg was uniform in diameter, we measured the maximum diameter of the conic tip and the peg of the two sensilla in each larva.

### Anterograde Staining of the Gustatory Receptor Neurons

The assembly of neurons from each of the two sensilla styloconica was labeled separately by cutting only one sensillum in each individual. Anterograde staining of the current receptor neurons was performed by cutting the tip of the sensillum using fine scissors. Next, crystals of fluorescent dye (tetramethylrhodamine dextran with biotin, Micro-Ruby, Molecular Probes; Invitrogen, Eugene) were applied to the cut end of the remaining organ via a micro needle. Then, the larval head was kept at 4°C for 24 h for allowing dye transportation in the neural axons. The preparation, including the brain and the SOG, was subsequently dissected out from the head capsule and fixed in 4% paraformaldehyde for 2 h at room temperature before being rinsed in phosphate-buffered saline (PBS; in mM: 684 NaCl, 13 KCl, 50.7 Na_2_HPO_4_, 5 KH_2_PO_4_, pH 7.4). Before being dehydrated and cleared in methylsalicylate, the preparations were stained by means of synapsin immunolabeling.

### Immunostaining for Identifying Neuropil Structures

For visualizing the neural architecture of the stained preparations, immunostaining with an antibody labeling presynaptic terminals was performed. The antibody SYNORF1 (Developmental Studies Hybridoma Bank, University of Iowa) was used as the primary antibody. The preparations were pre-incubated in 5% normal goat serum (Sigma, St. Louis, MO) in PBS containing 0.5% Triton X-100 (PBSX; 0.1 M, pH 7.4) for 3 h to minimize non-specific staining and then incubated in SYNORF1 at 1∶100 in PBSX at 4°C for 5 days. After rinsing in PBS for 6×20 min, the preparations were incubated in a Cy2-conjugated anti-mouse secondary antibody (Invitrogen, Eugene, OR; dilution 1∶300 in PBSX) at 4°C for 3 days. Finally, the preparations were rinsed 6×20 min in PBS, dehydrated with ascending ethanol series (50%, 70%, 90%, 96%, 2×100%, 10 min each), and mounted in methylsalicylate.

### Confocal Image Acquisition

Serial optical images of the preparations were obtained by using a confocal laser scanning microscope (LSM 710, META Zeiss, Jena, Germany) with a 10×(Fluar 10×/0.50), a 20×(Plan-Apochromat 20×/0.8), and a 40×(Plan-Neofluar 40×/1.3 oil DIC) objective. The rhodamine was excited by a 543 nm line of a HeNe1 laser and the Cy2 (from the immunostaining) by the 488-nm line of an Argon laser. The confocal images were obtained with the resolution of 1024×1024 pixels. All successfully stained preparations, i.e. 23, were scanned via the confocal microscope.

### Digital 3D-reconstruction of Neuropil Regions and Neurons

In order to visualize the stained axons and the surrounding neuropil regions, the confocal image stacks were reconstructed by means of the software AMIRA 4.1 (Visage Imaging, Fürth, Germany). The neuropil regions were reconstructed by using the segmentation editor and the neuron axons by using the skeleton module. Of the successfully stained preparations, 10 were reconstructed by means of the AMIRA software − five including stained afferents emanating from the medial sensillum and five from the lateral.

In order to compare the target areas of gustatory receptor neurons that originated from the medial and lateral sensilla styloconica, respectively, the reconstructed neurons and surrounding neuropils structures from individual preparations were brought into the same frame. This frame was made from the preparation being best labeled via the synapsin immunostaining technique. Briefly, the label images of the preparation with stained neurons were affine- and elastically registered to the corresponding label images of the frame. The resulting transformation parameters for the neuropil structures were subsequently applied to the reconstructed neurons. The results were then carefully evaluated by comparing the confocal images with the obtained model.

### Image Processing

The figures were edited in Adobe Illustrator CS3. Prior to editing, brightness and contrast of the images were adjusted in Photoshop CS3. The orientation of the brain and the SOG and naming of the neuropil structures are indicated as described by Kent and Hildebrand [32].

### Statistical Analyses

The size of the conic tip and the uniporous peg in the lateral and the medial sensillum were compared using a paired t-test. The significance level was set to *P*<0.05. All data were analyzed using SPSS software version 10.0.

## Results

### Morphology of the Maxillary Sensilla Styloconica

**Figure 1:**
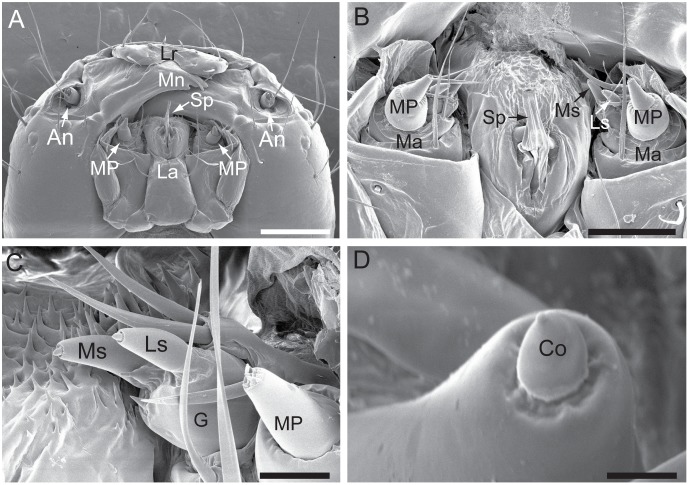
Morphologies of the two sensilla styloconica in the fifth instar larvae of *H. armigera*. (A) Ventral view of the head showing a pair of antennae (An), the labrum (Lr), the mandible (Mn), the labium (La), the maxillary palps (MP) on the maxilla, and the spinneret (Sp). (B) Close-up view showing the two sensilla styloconica, i.e. the lateral sensillum styloconicum (Ls) and the medial sensillum styloconicum (Ms) located on the maxilla (Ma). (C) Close-up view of the medial and the lateral sensillum styloconicum located on one galea (G). (D) Near view showing the socketed conic tip (Co) inserted into the large peg of a sensillum styloconicum. Scale bars: 500 µm in A, 200 µm in B, 50 µm in C, and 5 µm in D.

The chewing mouthparts of the 5^th^ instar *H. armigera* larvae are shown in Figure 1. On the galea of the maxilla, there are two uniporous sensilla styloconica, termed the medial sensillum and lateral sensillum, respectively (Fig. 1C). Each sensillum comprises a socketed conic tip inserted into the large peg (Fig. 1D). The peg size of the medial sensillum is significantly bigger than that of the lateral sensillum (*P*<0.05; Table 1). The size of the conic tip on the peg, however, is similar between the two sensilla styloconica (*P*>0.05).

**Table 1:** Length and diameter of the peg and the conic tip of each sensillum styloconicum.

	Uniporous peg^a^		Conic tip^b^	
	Length (µm)^c^	Diameter (µm)^d^	Length (µm)^c^	Diameter (µm)^d^
Medial sensillum	69.51±0.93	32.06±0.52	7.06±0.12	5.93±0.07
Lateral sensillum	59.56±1.23	30.42±0.53	7.01±0.09	5.99±0.11
*P* value^e^	<0.001	0.001	0.65	0.66

A paired sample t-test is used to determine whether there is a significant difference of size between the medial sensillum and the lateral sensillum of *H. armgiera* larvae (*P*<0.05).

a The uniporous peg is the main peg of each sensillum.

b The conic tip is the socketed tip on the main peg of the sensillum.

c The lengths (mean ± SE) represent the length of the peg and the conic tip, respectively (the length of the peg does not include the conic tip).

d The diameters (mean ± SE) represent the diameter of the peg and the conic tip, respectively.

e Probability of difference of the mean length or the mean diameter between the medial sensillum and the lateral sensillum in a column. The current lengths and diameters were measured in one side of the mouthparts in each single larva, N = 30.

### Anatomy of the Larval Brain and Suboesophageal Ganglion

**Figure 2:**
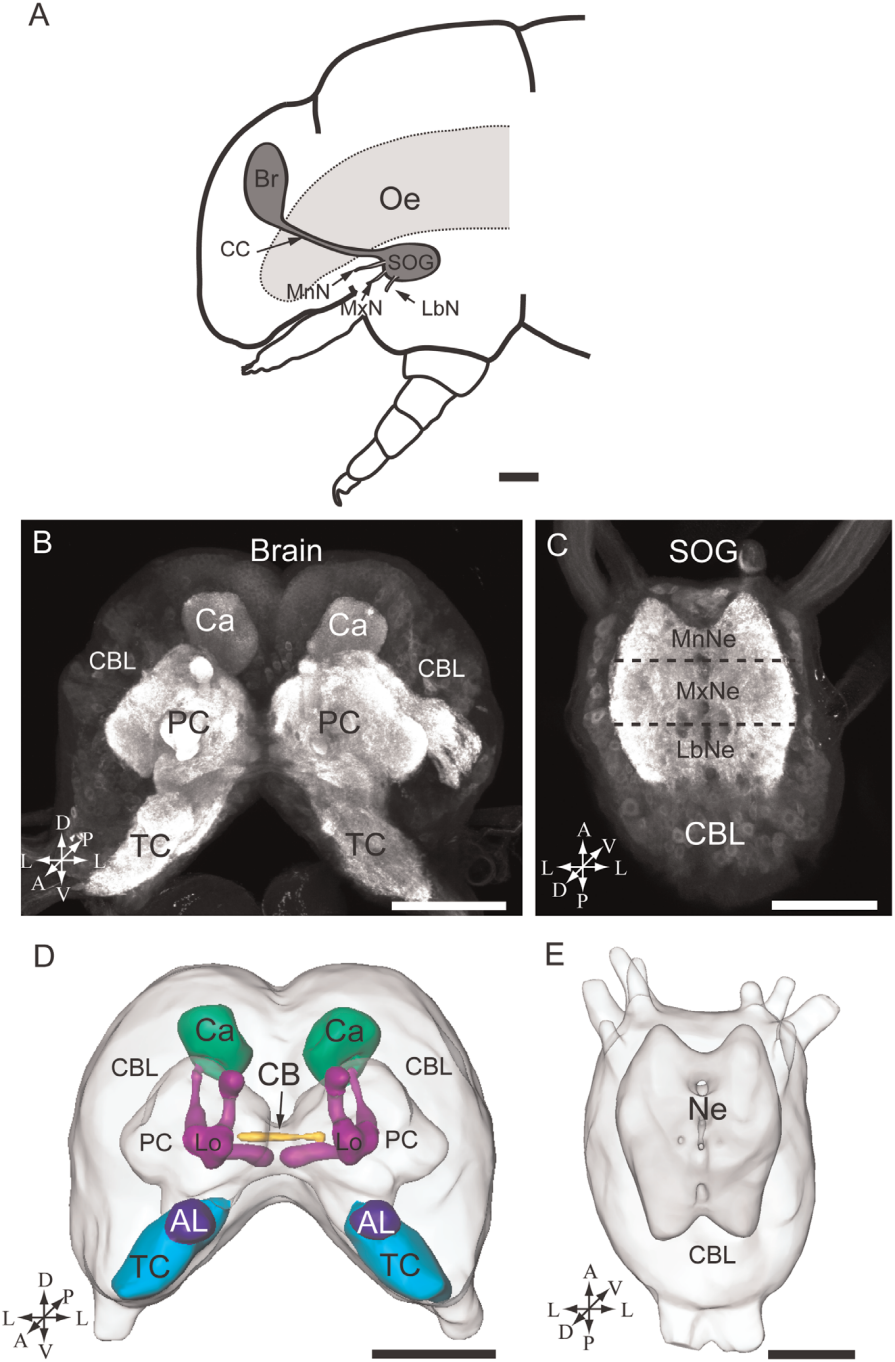
The brain and the suboesophageal ganglion of the fifth instar larvae of *H. armigera*. (A) Diagram of the larval head including the brain (Br) and the suboesophageal ganglion (SOG). (B) Confocal image of the brain showing the cell body layer and the central neuropils. (C) Confocal image of the SOG showing the cell body layer (CBL) and the three fused neuromeres, from anterior to posterior: the mandibular neuromere (MnNe), the maxillary neuromere (MxNe), and the labial neuromere (LbNe). (D) 3D reconstruction of the whole brain. (E) 3D reconstruction of the SOG. A anterior, D dorsal, L lateral, M medial, P posterior, V ventral. Oe oesophagus, CC circumoesophageal connectives, MxN maxillary nerve, LbN labial nerve, MnN mandibular nerve, TC tritocerebrum, PC protocerebrum, Ca calyces of the mushroom body, AL antennal lobe, CB central body, Lo lobles, Ne neuromere. Scale bars: 250 µm in A, 100 µm in B, C, D, and E.

As illustrated in figure 2A, the head capsule of the 5^th^ instar larva of *H. armigera* comprises the supraoesophageal ganglion (the brain) and the SOG. The two ganglia make an angle of approximately 90 degrees to each other and are linked by a pair of long circumoesophageal connectives (Fig. 2A). Each ganglion contains an outer shell of neuronal cell bodies and a central fibrous neuropil. The central neuropil of the brain consists of three neuromeres, i.e. the tritocerebrum, the deutocerebrum (the antennal lobes), and the protocerebrum (Fig. 2B, D). Within the protocerebrum, the prominent neuropil regions include the central body and the paired mushroom bodies, i.e. the calyces, the pedunculus, and the lobes (Fig. 2B, D).

The SOG is also a fusion of three neuromeres (Fig. 2C, E). From anterior to posterior, these include the mandibular neuromere, the maxillary neuromere, and the labial neuromere, each being linked to the corresponding segmental appendage via the mandibular, the maxillary, and the labial nerve, respectively (Fig. 2A, C).

### Central Projections of Gustatory Receptor Neurons from the Sensilla Styloconica

**Figure 3:**
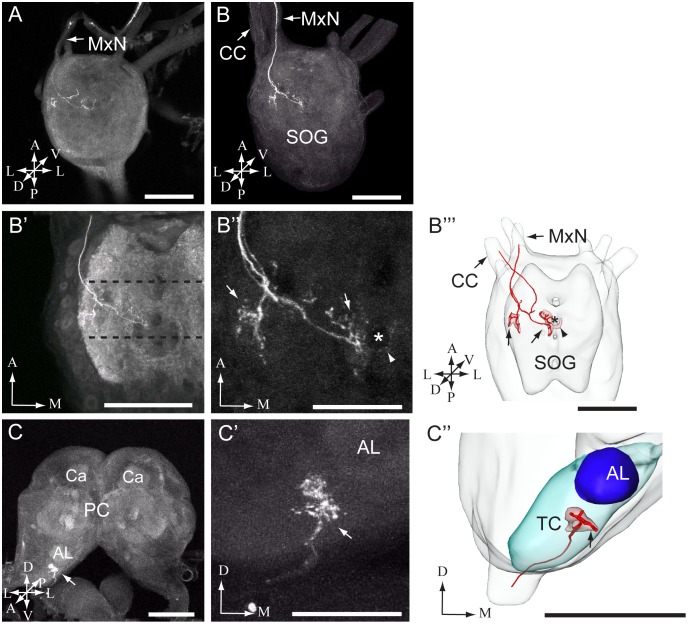
Images of the larval suboesophageal ganglion and brain with stained afferents originating from the medial sensillum. (A, B) Confocal images from two preparations showing axon projections in the suboesophageal ganglion (SOG). In A, a preparation containing two labeled axon is presented, and in B, another preparation containing three stained fibers. As indicated by arrows in both images, the axons entered the SOG via the maxillary nerve (MxN). In B, the arrow to the left points to the circumoesophageal connective (CC). (B’-B’’) High resolution images of axon terminals in the SOG can be seen (same preparation as in B). In B’, the target region of the terminals, i.e. the maxillary neuromerebeing situated between the dotted lines is shown. In B’’, the detailed pattern of the axon terminals giving off processes in the ventro-lateral (left arrow) and the dorso-medial (right arrow) neuromere are shown. The arrowhead points to a few arborizations crossing the midline and the asterisk indicates a tract in the SOG, which was not stained by the synapsin antibody. (B’’’) 3D AMIRA-reconstruction of the SOG including target regions (indicated by red) of axons originating from the medial sensillum (arrows; made from the preparation shown in B-B’’). The stained axons in the MxN and the CC are indicated by single lines in the AMIRA model. (C-C’) Confocal images showing the axon terminals in the brain (same preparation as presented in B-B’’). In C, the arrow points to the innervated brain region, i.e. the tritocerebrum (TC). In C’, the stained axon terminals in the TC are shown in a closer view. (C’’) 3D AMIRA reconstruction of the TC including the target region (arrow) of the stained axons (made from the preparation presented in C-C’). The stained axons entering the TC are indicated by one single line in the AMIRA model. AL antennal lobe, Ca calyces, PC protocerebrum. A anterior, D dorsal, L lateral, M medial, P posterior, V ventral. Scale bars: 100 µm in A, B, B’, B’’’, C, and C’’; 50 µm in B’’ and C’.

**Figure 4:**
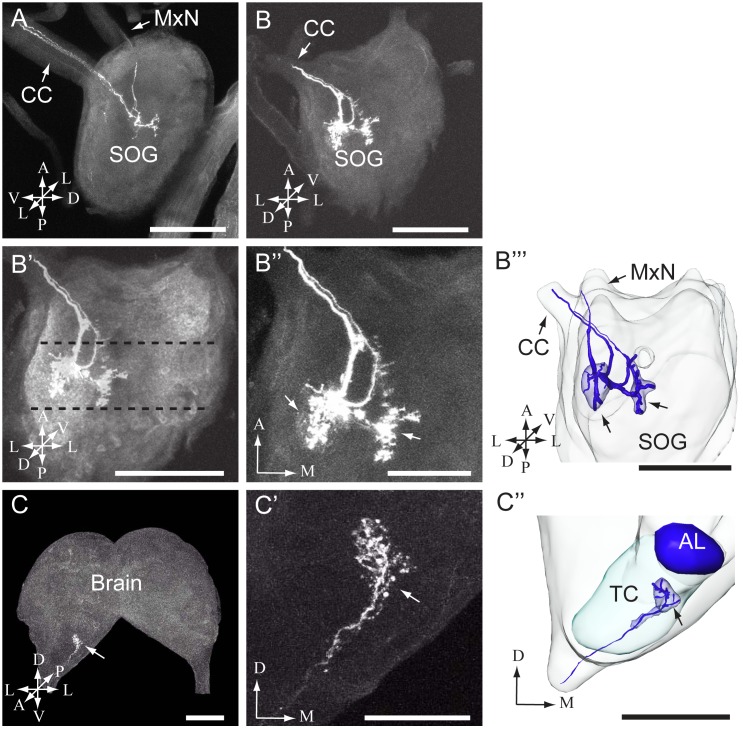
Images of the larval suboesophageal ganglion and brain with stained afferents originating from the lateral sensillum. (A-B) Confocal images from two different individuals showing stained projections in the subesophageal ganglion (SOG). In A, one preparation containing three labeled axons is shown, and in B one preparation containing two stained axons. The axons entered the SOG and projected further to the brain via the circumoesophageal connective (CC). (B’-B’’) High resolution images showing the projection pattern in the SOG (same preparation as presented in B). In B’, the target region of the axon terminals, i.e. the maxillary neuromere of the SOG, being situated between the dotted lines, is shown. In B’’, the two distinct regions being innervated are shown (arrows). (B’’’) 3D AMIRA-reconstruction of the SOG including the two regions targeted by the stained axons originating from the lateral sensillum (arrows). The AMIRA reconstruction originates from the preparation presented in B. The stained axons in the MxN are indicated by one single line in the AMIRA reconstruction. (C-C’) Confocal images showing terminals of the stained axons within the tritocerebrum (TC) of the brain (different preparation from those presented above). In C, the arrow points to the stained branches in the TC. In C’, the arborizations are shown at a higher magnitude. (C’’) 3D AMIRA-reconstruction of the tritocerebrum (TC) including the target region of the stained neurons originating from the lateral sensillum (arrow). The AMIRA reconstruction originates from the preparation presented in C. The stained axons entering the TC are indicated by one single line in the AMIRA reconstruction. AL antennal lobe, A anterior, D dorsal, L lateral, M medial, P posterior, V ventral. Scale bars: 100 µm in A, B, B’, B’’’, C, and C’’; 50 µm in B’’and C’.

**Figure 5:**
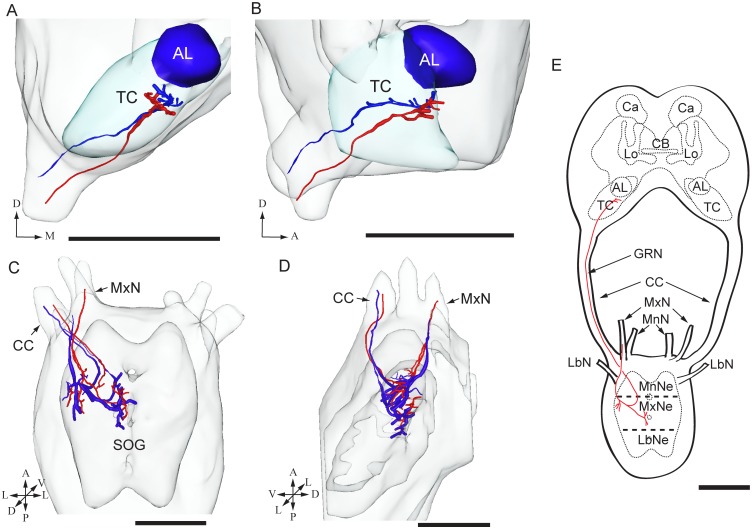
3D-reconstructions and schematic representation showing the target regions of stained axons from both sensilla styloconica. The reconstructed axons in this figure are the same as those presented in Fig. 3 and 4. (A-B) 3D AMIRA-reconstractions of the dorso-anterior tritocerebrum (TC) in a frontal (A) and lateral view (B) showing the terminal regions of projections originating from the medial (red) and the lateral (blue) sensillum. (C-D) 3D AMIRA-reconstructions of the suboesophageal ganglion (SOG) in a dorsal (C) and lateral view (D) showing the terminal branches of projections emanating from the medial (red) and the lateral (blue) sensillum. As demonstrated in the model, the axon assemblies from both sensilla, which enter the SOG via the maxillary nerve (MxN; arrow) and exit via the circumoesophageal connective (CC; arrow), target overlapping regions in the current ganglion. (E) Schematic representation of the gustatory projections associated with the MxN. As shown in the drawing, the assembly of afferents gives off arborizations in the ipsilateral maxillary neuromere of the SOG before ascending to the brain via the CC, terminating in the TC. MnNe mandibular neuromere, MxNe maxillary neuromere, LbNe labial neuromere, MnN mandibular nerve, LbN labial nerve, GRN gustatory receptor neurons. AL antennal lobe, Ca calyces, CB central body, Lo mushroom body lobes. A anterior, D dorsal, L lateral, M medial, P posterior, V ventral. Scale bars 100 µm.

In total, dye was applied to one of the two sensilla stylochonica in 130 preparations. Of these, 23 were successfully stained −11 preparations showing labeling of projections arising from the medial sensillum and 12 from the lateral. Due to the dense staining pattern formed by the axons it was not possible to discriminate between the central projections of individual neurons originating from the same sensillum. The typical projection patterns of the neuron groups housed by the medial and the lateral sensillum are shown in figure 3 and figure 4, respectively. As demonstrated in these figures, the general projection patterns occurring from the assembly of labeled neurons housed by each of the two sensilla seem to be similar. In figure 5, the overlapping projection patterns of these afferents are demonstrated by reconstructed models of the stained projections originating from each sensillum being positioned into the same reference framework.

Thus, the stained axons of both sensilla enter the maxillary neuromere of the SOG via the ipsilateral maxillary nerve (MxN; Figs. 3, 4, and 5). In the SOG, the axons give off arborizations in the ventro-lateral and the dorso-medial neuromere on the ipsilateral side (Fig. 3 A, B-B’’’, Fig. 4 A, B-B’’’, Fig. 5 C–E). From the SOG, the sensory fibers extend into the tritocerebrum via the circumoespohageal connectives (Figs. 3, 4, and 5). The confocal images in figure 4 B-B’’’ show one thick and one thin axon projecting from the SOG via the circumoespohageal connectives. In the tritocerebrum, the axon terminals target an ipsilaterally dorso-anterior region located ventro-laterally of the antennal lobe (Fig. 3 C-C’’, Fig. 4 C-C’’, Fig. 5 A, B, and E). The entire confocal stacks from which figure 4B and figure 4C’ were made are shown as Movie S1 and Movie S2, respectively, in the Supporting Information. As concerns the similar projection patterns formed by the groups of stained axons arising from the two sensilla, it should be mentioned that a small detail applying to the afferents of the medial sensillum only was observed, namely a few arborizations crossing the midline to the contralateral side of the SOG (Fig. 3B’’, B’’’, arrowhead). The majority of the successfully stained preparations contained two to three labeled neurons. More precisely, eight preparations showed two stained neurons, seven preparations three stained neurons, three preparations four stained neurons, and two preparations one and five stained neurons, respectively. Examples of the most frequent staining pattern observed, i.e. two and three labeled axons, are included in figures 3 and 4.

## Discussion

### Morphology of the Maxillary Sensilla Styloconica

By using electron microscopy, we described the ultrastructure of the sensilla styloconica of the last instar larvae of *H. armigera*. Generally, their morphology including a socketed conic tip inserted into the large peg was similar to that of corresponding sensilla formerly described in other lepidopteran species [5], [7], [9]–[12], [14], [15], [17], [18]. The significant difference in size between the pegs of the two sensilla styloconica, as found here, has not been reported previously, however. It has been stated that the medial senillum and the lateral sensillum of *H. armigera* larvae respond to different stimuli [11], [47], [49]–[51]. Whether this distinction of morphology is due to a difference in gustatory function is an open question.

### Identification of the Stained Axons and Comparison with Other Lepidopteran Larvae

The central projection patterns formed by the group of stained neurons housed within each of the two sensilla appeared similar. As each sensillum probably contains four gustatory receptor neurons and one mechanosensory, we cannot definitively determine the identity of the individual axons stained. However, in all preparations including two or more stained fibers, we can ascertain that there are one or several gustatory afferents present (Fig. 3B’’ and Fig. 4A, B-B’’). Furthermore, based on the current findings showing similar projection patterns for the two groups of stained neurons originating from the medial and the lateral sensillum, respectively, it seems reasonable to suppose that they carry information about the same sensory modality − namely, taste.

The staining experiments showed that the sensory neurons project via the maxillary nerve to the ipsilateral maxillary neuromere of the SOG, where numerous neural branches are given off before the axons pass further on to the tritocerebrum via the ipsilateral circumoesophageal connective. This projection pattern is similar to that reported for corresponding neurons in larvae of *A. yamamai* [33] and *M. sexta* [32]. Of the two previously studied species, there are subtle differences regarding the projection pattern in the SOG, however. When comparing the projection patterns of *M. sexta* larvae displaying axon arborizations in the ventrolateral region of the maxillary neuromere and that of *A. yamamai* larvae showing projections in the anterior half of the maxillary neuromere, near the midline, it is obvious that the current findings are most similar to those of *M. sexta*. As none of the previous studies described the distinct projection pattern in the tritocerebrum, we cannot compare this part of our data with those of the other species. Interestingly, gustatory afferents passing in the maxillary nerve of *Drosophila* larvae are also reported to innervate an ipsilateral region of the SOG [36].

### Comparison of the Gustatory Pathways in the Heliothine Larva and the Adult

The SOG and brain of lepidopteran larvae are separated while the current ganglia are tightly fused in adults [4]. Nevertheless, the projection pattern of axons housed by the sensilla styloconica of *H. armigera* larvae, as presented here, is partly similar to the pattern of taste afferents in adults of the closely related species, *H. virescens*. Thus, in the adult, taste neurons originating from the sensilla styloconica on the proboscis also enter the SOG via the MxN [52], [53]. In addition, these gustatory afferents are reported to target a dorsal region of the ipsilateral SOG/tritocerebrum, similarly to the present findings [52], [53]. Some additional axons originating from the proboscis were reported to target bilateral regions located anteriorly in the SOG [52]. We found no bilateral projections in the *H. armigera* larvae. The presence of exclusively ipsilateral projections in larvae of the other studied lepidopteras, as well, i.e. *A. yamamai* [33] and *M. sexta* [32], suggests that there is a difference in the current gustatory pathway between larvae and adults.

### Comparison of the Current Gustatory Pathways with Those of Non-lepidopteran Insect Species

Similarly to the present findings, axons of gustatory receptor neurons entering the SOG via the MxN have been reported in several non-lepidopteran insect species. In the locusts, *Locusta migratoria* and *Schistocerca gregaria*, axons originating from the maxilla also enter the SOG via the MxN, and then project to the tritocerebrum via the circumoesophageal connective [49]. In the honey bee, *Apis mellifera*, gustatory receptor neurons on the proboscis project to the maxillary neuronmere of the SOG/tritocerebrum via MxN [54]–[56]. Furthermore, in fly adults, some gustatory axons projecting from the maxillary palps via the MxN fuse with the labial nerve and proceed to the SOG [4], [50], [57], [58]. Finally, in the African malaria mosquito, *Anopheles gambiae*, and the yellow fever mosquito, *Aedes aegypti*, axons from sensilla located on the maxillary palps project into a posterodorsal region of the SOG via the maxillary-labial nerve [51], [59].

### Concluding Remarks

Our data suggest that the assemblies of gustatory neurons housed by each of the two sensilla styloconica located on the maxillary galea of *H. armigera* larvae target similar regions in the central nervous system. Furthermore, our anatomical data on the central projections from the current sensilla are in correspondence with previous studies of other lepidopteran larvae, suggesting a conserved gustatory coding mechanism among different species. Furthermore, the similarities between the gustatory pathway presented here and parts of those possessed by the adult insect may indicate a persistence of particular taste neurons during metamorphosis. In future investigations, biologically relevant taste stimuli should be tested during intracellular recordings from single gustatory neurons of both heliothine larvae and adults. Such experiments will provide more detailed information about the so far relatively unexplored mechanisms characterizing information processing in the central gustatory pathways, not only of moths but of insects in general.

## Supporting Information

Movie S1 **Movie of a confocal stack showing the projection pattern of stained axons in the subesophageal ganglion (SOG) (ventral view).** Two stained fibers originating from the lateral sensillum give off processes in the ipsilateral SOG before projecting into the circumoesophageal connective. The confocal stack includes the image shown in Fig. 4B (For further details, see legend to Fig. 4).
(MPG)

Movie S2 **Movie of a confocal stack showing the projection pattern of stained axons in the tritocerebrum (frontal view).** The confocal stack includes the image shown in Fig. 4C’ (For further details, see legend to Fig. 4).
(MPG)
